# Dietary and Cosmetic Antioxidants

**DOI:** 10.3390/antiox13020228

**Published:** 2024-02-13

**Authors:** Irene Dini

**Affiliations:** Department of Pharmacy, University of Naples Federico II, Via Domenico Montesano 49, 80131 Napoli, Italy; irdini@unina.it

Spices, herbs, fruits, whole grains, vegetables, and sea organisms contain antioxidant molecules that can scavenge free radicals and reduce their development, quenching the reactive oxygen and nitrogen species [1]. Even agri-food waste can contain bioactive compounds with antioxidant, anti-inflammatory, and antimicrobial activities [2], which could be exploited to formulate natural and sustainable cosmetics; in this industry, the skin’s well-being and environmental problems are two inextricably linked realities [3].

This Special Issue includes research articles and reviews addressing bioactive compounds in food and organic waste with potential hydrating, protective, repairing, regenerating, and whitening effects on the skin and delivery systems capable of improving their performance in cosmetics.

Dini reviewed the potential for nanotechnologies, in supplements and cosmetics formulations, to enhance the performance of nutricosmeceutic products.

Gu et al. studied the potential for alginate–chitosan-coated nanoliposomes to improve the bioavailability of bamboo leaf flavonoids. Moccia et al. investigated transfersomes’ efficacy as carriers of the ellagic acid obtained from a chestnut-wood mud industrial byproduct. Dini and Mancusi reviewed the potential role of biopeptides as antimicrobial, antioxidant, antiaging, and anti-inflammatory molecules. Kirindage et al. examined the antioxidative and anti-inflammatory actions of fucosterol extracted from the brown algae *Sargassum horneri.* Mansinhos et al. evaluated the whitening and sun-protective potential of Thymus lotocephalus extracts. Gigliobianco et al. investigated pomegranate and its waste products’ phenol components profile, cell viability, and antioxidant and antibacterial activities. Yang et al. determined the possibility of developing cosmetic materials with skin-whitening and anti-inflammatory functions using fermented maca root extracts with Lactobacillus strains. Aiello et al. studied the effect of carnosine on UVA-induced changes. Yang et al. investigated the ability of an enzyme (carbohydrase celluclast) to improve the cosmeceutical potential of *Ishige okamurae*. Cardeira et al. evaluated the potential anti-inflammatory and antimicrobial activity of protein-rich extracts from sardine waste and codfish frames. Choi et al. assessed the skin-whitening action of the nomilin extracted from discarded yuzu byproducts. Finally, Mahendra et al. reviewed the anti-inflammatory, antioxidant, antibacterial, wound-healing, and skin-whitening potential of *Swietenia macrophylla*.

The articles in this Special Issue confirm the strong potential of foods (vegetable and animal) and organic waste as sources of bioactive compounds with a lightening, moisturizing, and protective action on the skin and as preservatives to increase the shelf life of cosmetics. They examine nanotechnologies to improve the bioavailability of bioactive compounds, ensure their release at the site of action, cover their unpleasant taste, and extend their expiry date. Nevertheless, the authors agree that there is a need for further toxicology studies to guarantee consumer safety and for experimental research on the large-scale recovery of bioactives from organic matrices before new cosmetics can be developed.
